# Highly reactive, liquid diacrylamides via synergistic combination of spatially arranged curing moieties

**DOI:** 10.3762/bjoc.13.40

**Published:** 2017-02-27

**Authors:** Maximilian Maier, Magnus S Schmidt, Markus Ringwald, Christoph P Fik

**Affiliations:** 1 Dentsply Sirona Restorative, De-Trey-Str. 1, 78467 Konstanz, Germany; 2 MCAT GmbH, Raiffeisenstr. 35, 78166 Donaueschingen, Germany

**Keywords:** acrylamide, allyl, cyclopolymerization, photopolymerization, spatial effect

## Abstract

Six polymerizable *N*,*N*’-diacylamides containing spatially arranged *N*-acryl, *N*-allyl and/or *N*-alkyl groups were prepared via two-step syntheses and characterized by ^1^H/^13^C NMR-spectra, refractive index (RI) and viscosity measurements. Photo DSC measurements on activated samples provided reactivity parameters ∆*H*_p_, *R*_p,max_ and *t*_max_, while FTIR spectra before and after curing elucidated the underlying polymerization mechanism. Mechanical testing of the obtained polymers exhibited gradual differences in network densities, depending on the intramolecular arrangement and number of functional groups. Overall, a general building principle for highly reactive, liquid diacrylamides via synergistic combination of optimally arranged functional groups could be identified. The highest possible level of intramolecular synergism was found for low viscous *N*,*N*'-diacryloyl-*N*,*N*'-diallyl-1,4-but-2-enediamine.

## Introduction

The selection of suitable monomers is a critical step for any free-radical polymerization approach. Particularly for (in situ) photo-induced polymerizations, monomers should comprise sufficient solubility in a given matrix, moderate viscosity, matching refractive indices as well as an optimized reactivity – the proper design of these features ensures continuous light transmittance, adequate propagation rates and, ultimately, thorough polymerization [1–2]. The number of applications for UV–vis curable monomer systems has greatly increased over the last decades [3]. At the same time, the selection of new monomers and crosslinkers remained limited [4].

Mono-, di-, tri- and multifunctional (meth)acrylates are among the first choices for photopolymerized mixtures as they exhibit a favorable balance between reactivity and thermal stability upon storage [5–7]. Moreover, they comprise compatibility with different matrices/solvents together with an adequate reactivity in a broad temperature range [8–10]. In general, acrylate monomers exhibit a higher reactivity than the respective methacrylates [11–13], but tend to be more sensitive to oxygen inhibition [14]. A major drawback of many (meth)acrylate-based compositions, however, is their susceptibility to premature hydrolysis when used in aqueous solutions, especially at pH values <2.5 [15–16].

One strategy to improve the hydrolytic stability is the oxygen-to-nitrogen substitution. The obtained class of (meth)acrylamides is of interest in the field of biomedical applications, e.g., for dental materials, artificial cornea, or drug-delivery systems, for which contact with body fluids is inevitable [17–18]. Whilst some of the resulting secondary di(meth)acrylamides end up being solids, tertiary di(meth)acrylamides can be obtained as relatively low viscous, highly soluble/compatible liquids [19]. Furthermore, acrylamides are generally more reactive than the respective methacrylamides. Regarding the substitution pattern, N-monosubstituted acrylamides tend to homopolymerize more readily than their N,N-disubstituted analogues [20]. Yet, acrylamides are particularly affected by the solvent regarding propagation reaction in free radical polymerization, even more so, if water is present [21].

Factors such as hydrogen bonding, hydrogen abstraction and the overall electronic characteristics are crucial in the design of improved monomer structures [22]. In this sense, Bowman et al. demonstrated increased photo-polymerization rates for monoacrylates equipped with secondary functionalities, yet limiting discussion to oxygen-based (meth)acrylate derivatives [23].

In this study, we present the synthesis and characterization of tailor-made, liquid *N*,*N*’-diacyl diacrylamides with enhanced reactivity through synergistic combination of spatially arranged curing moieties. The obtained structures were investigated in terms of underlying building principle, chemical and physical properties as well as polymerization behavior upon photoinitiation.

## Results and Discussion

**Scheme 1:**
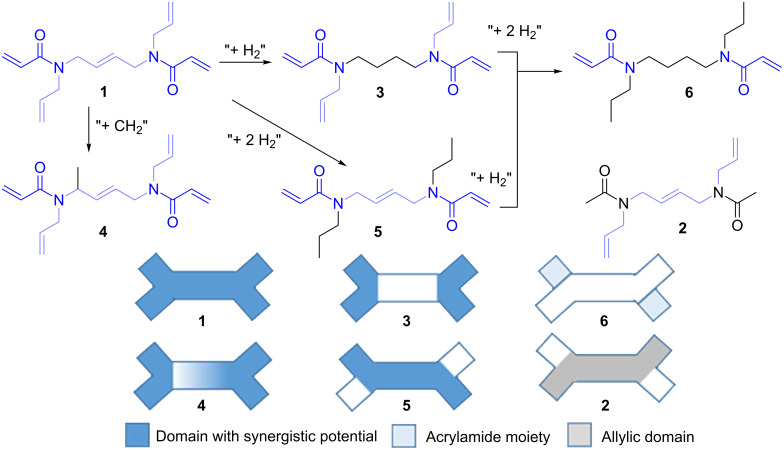
Top: Overview of the synthesized crosslinkers **1–6** and their correlation to each other via formal reactions. Bottom: Schematic of **1–6** in terms of their structural synergistic potential due to adjacent acrylamide and allyl functions.

As stated earlier [24] we strive to investigate the unique physical properties and reactivity of tertiary *N*,*N*’-diallyl-diacrylamides. Closely related to this class of crosslinkers are bifunctional *N*-alkyl-*N*-allylacrylamides, which are known to undergo radical cyclopolymerization due to their adjacent double-bond functionalities [25–27]. The propagation reaction of these structures proceeds intramolecularly between acryl and allyl groups and intermolecularly (mostly) between polymer-radical and acrylamide groups. Cyclo- is preferred over linear polymerization due to the preformed five or six-membered lactams and gets even more predominant with increasing chain length of *N*-alkyl groups [28]. Expanding this concept in view of an optimized spatial layout, we synthesized molecules with additional “internal” (at the molecules’ center), symmetrical allyl functions, connecting two *N*-allylacrylamide groups, thus adding a two-way, intramolecular reaction site. In order to individually assess the effect of “internal” and “external” (at the molecules’ periphery) *N*-allylic functions on the physical/polymerization properties, a systematic variation of the molecular structure has been realized. When allyl- and acrylamide functionalities were spatially adjacent, a “synergistic potential” beneficial in radical polymerization was expected (Scheme 1).

### Synthesis

**Scheme 2:**
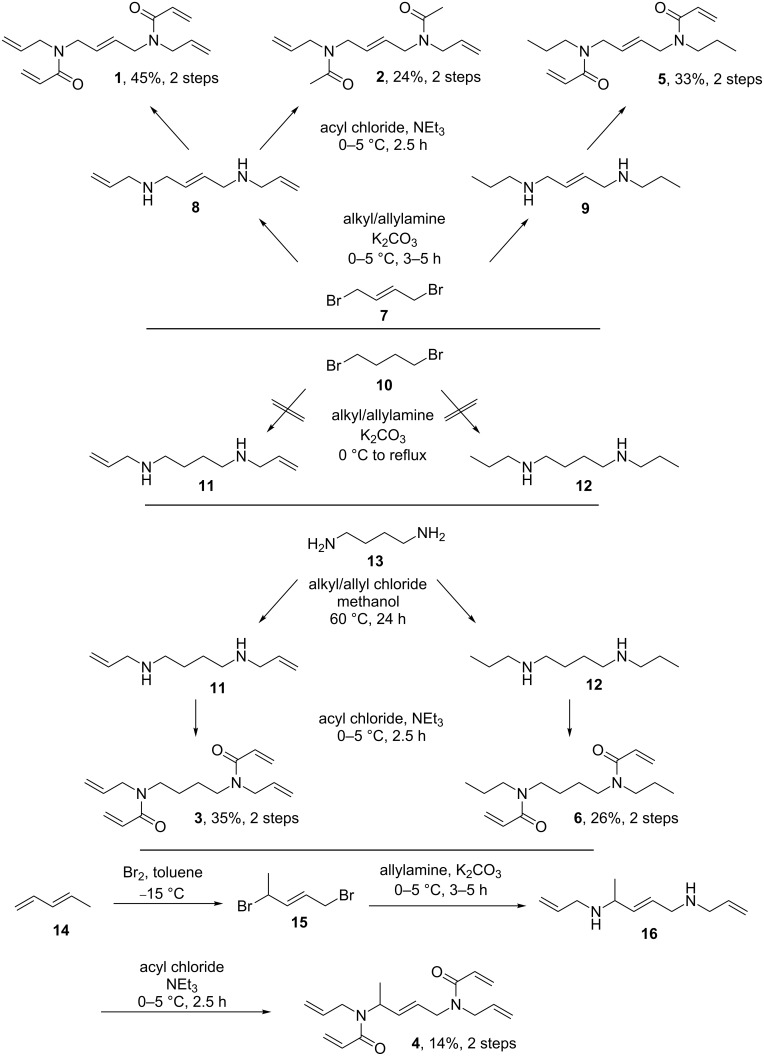
Synthetic pathways to structurally related compounds **1**–**6**.

**Scheme 3:**
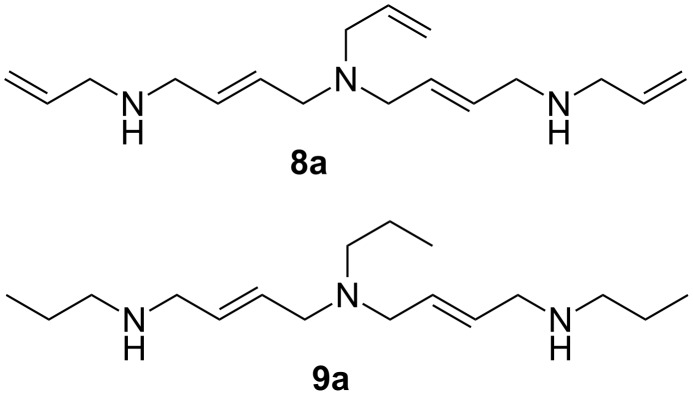
Byproducts **8a** and **9a**.

Six derivatives of highly functionalized crosslinkers **1**–**6** were synthesized as outlined in Scheme 2. We started from dibromide **7** to gain access to the corresponding compounds **1**, **2** and **5**. In case of the alpha-methyl compound **4**, we started from *trans*-1,3-pentadiene (**14**) and synthesized the dibromide **15** according to the work of Heasley et al. [29]. Intermediates like compound **8** have already been synthesized in the 1990s by Havis et al. through the dropwise addition of **7** in chloroform to a solution of primary amines such as aminocyclohexane at room temperature [30]. After stirring for 24 hours the resulting diamino hydrobromide was isolated in moderate yield. To avoid quenching/scavenging of the hydrobromide in the later stages, we decided to use a procedure which would allow the isolation of the free diamine. Therefore, we used a substantial excess of the alkylamine without any other/further solvent and potassium carbonate as scavenger base for the hydrobromide (leading to insoluble potassium bromide). After work-up, we could isolate crude diamines **8** and **9**, containing significant amounts (10–15%) of the tertiary amines **8a** and **9a** (Scheme 3) or diamine **16**, respectively; each could be used without further purification. Classical acylation with acetyl chloride or acryloyl chloride in the presence of triethylamine led to the corresponding diallyl diacylamides **1**, **2** and **5** in 24–45% yields or, in the case of the alpha-methyl-substituted system, to compound **4**, in 14% yield. In all systems, we were able to remove acylated byproducts of **8a** and **9a** by washing the organic solutions several times with 2 N HCl after which the compounds could be used without further purification. The synthesis of diamines **11** and **12** on the other hand was not possible by reacting 1,4-dibromobutane (**10**) with the corresponding alkyl or allylamines due to the lower reactivity of **10** compared to the unsaturated dibromide **7**.

Feuer et al. reported the synthesis of **12** and other similar derivatives through a multistep reaction with the final step comprising the treatment of *N*,*N*'-dipropylperhydropyridazine-3,6-dione with a borane solution in THF [31]. Considering the high reactivity of **7** and the assumption, that the allylic double bond to the halide is responsible for this effect, we decided to use 1,4-diaminobutane (**13**) and allyl chloride for the synthesis of diamine **11**. This also resulted in the positive side effect, that a formation of tertiary amines, comparable to compounds **8a** and **9a**, is not possible in this case. Interestingly, during the addition of allyl chloride to **13**, we could not observe any exothermic behavior or fast formation of the desired compound. So we decided to raise the temperature to 60 °C for 24 hours resulting in a significant increase of product formation. The subsequent direct acylation of the crude product **11** with acryloyl chloride afforded **3** in 35% yield. Based on this result, the analogous reaction of propyl chloride with 1,4-diaminobutane resulted in compound **6** in 26% yield.

**Scheme 4:**
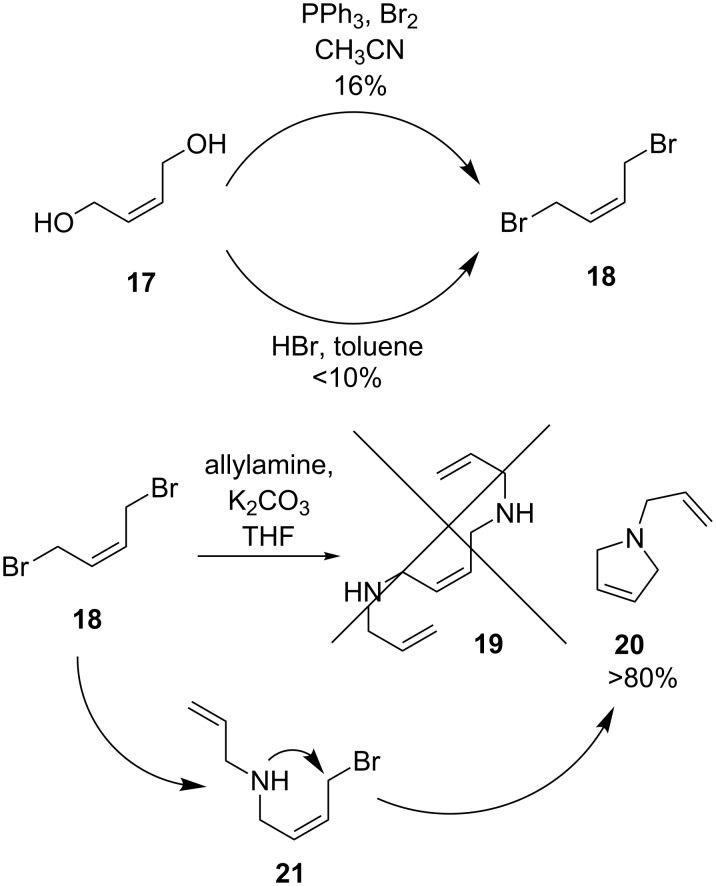
Synthetic pathways towards the planned *cis*-intermediate **19**.

In this context, attempts were made towards a cost-efficient synthesis of a possible *cis*-compound **19** (Scheme 4). For this, *cis*-dibromobut-2-ene (**18**) was synthesized from *cis*-but-2-ene-1,4-diol (**17**) using two different pathways; both reactions resulted in poor yields and product quality. Unfortunately, the reaction of **18** with allylamine did not result in the formation of **19**, but to the undesired cyclic compound **20**. The formation of the latter compound can only be explained by an intramolecular reaction of **21** (Scheme 4).

**Scheme 5:**
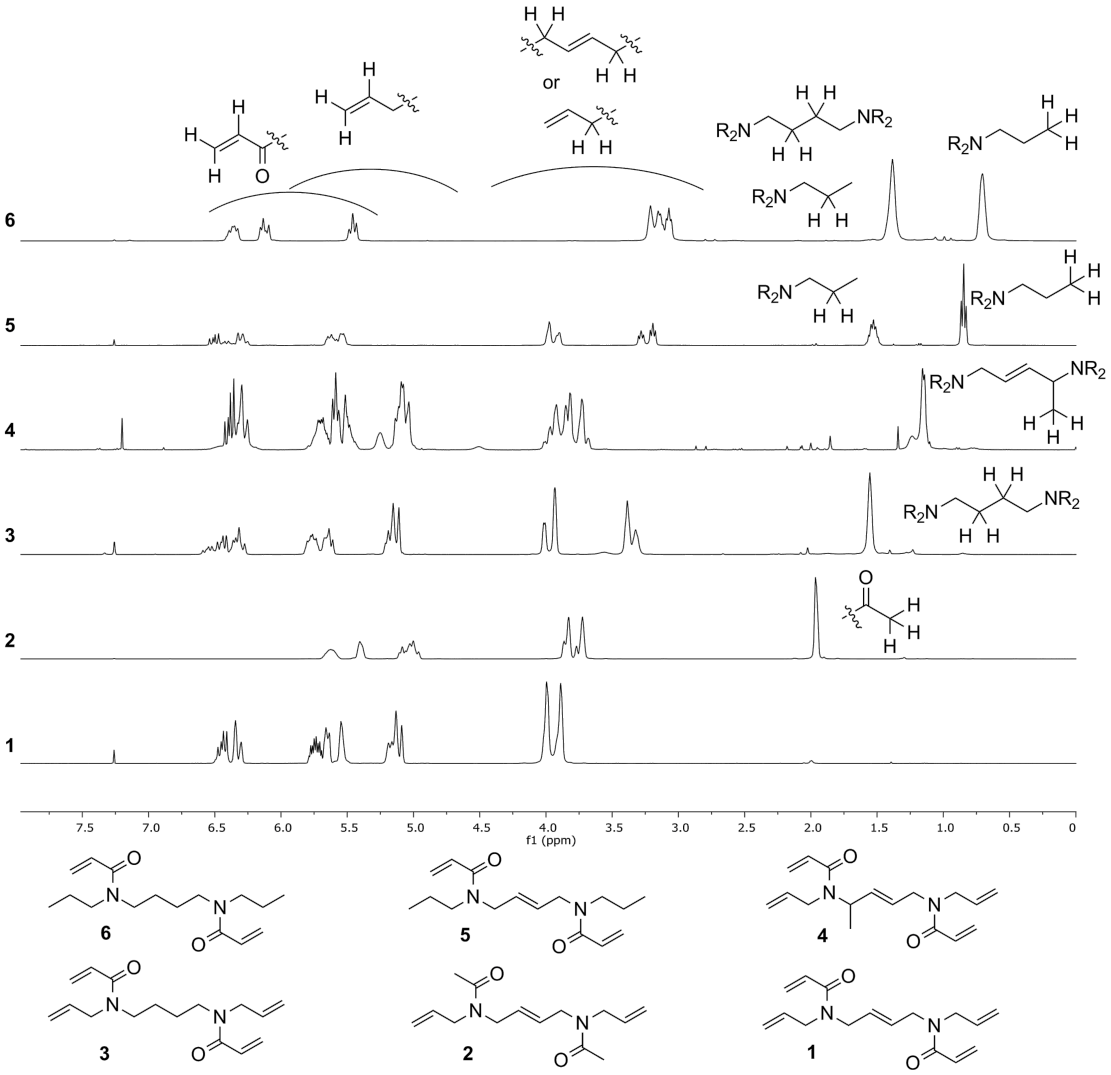
Comparison of structural elements of **1–6** in the ^1^H NMR spectra (400 MHz).

The NMR spectra of the final compounds show interesting aspects, reflecting the similar characteristic for this family of compounds (Scheme 5). Most importantly, all compounds exhibit line broadening (^1^H NMR) or multiple signal sets (^13^C NMR) for the possible *E*/*Z*-rotaisomers resulting in doubled signal sets in the ^1^H and ^13^C NMR spectra. The broader spectral field of the ^13^C spectra as well as the used decoupled method provided generally high resolution, led to hardly any overlap of the signals and resulted in the observed multiple signal sets. In the ^1^H NMR spectra, however, the small differences of the chemical shifts for the different rotaisomers lead to a decrease of the resolution. This makes it quite demanding to read out any coupling constants, as broad multiplets for the many methylene and double bond protons are observed. Nevertheless, the ^1^H NMR spectra (in combination with the two-dimensional methods COSY and HSQC) provided significant information for the classification of the compounds (Scheme 5).

**Figure 1:**
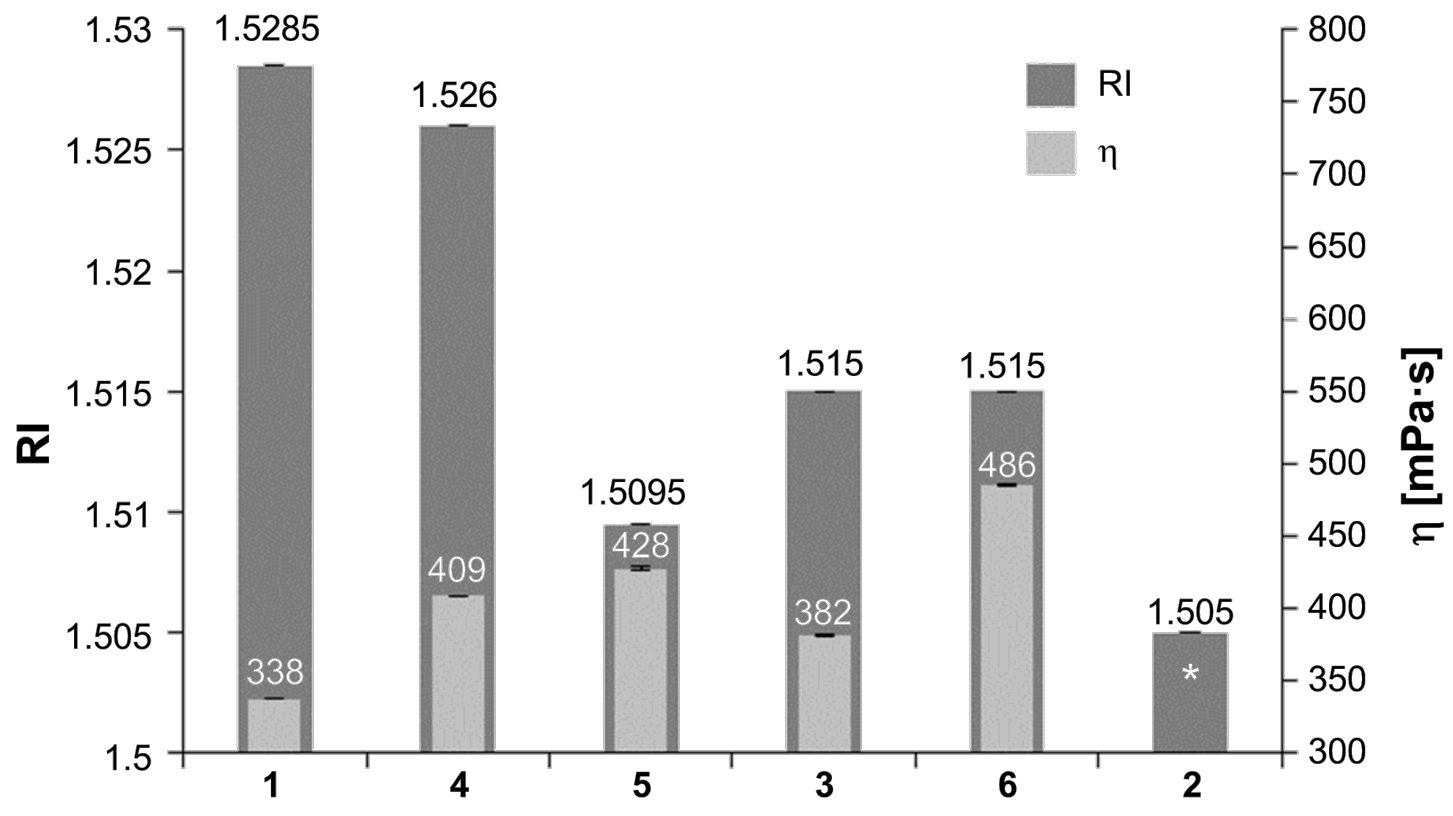
Refractive indices (RI) and viscosities (η) of crosslinkers **1–6** (* solid at room temperature).

Due to the increasing number of double bonds, refractive indices (RI) n^D^_20_ ranged from 1.505 to remarkable 1.529. RIs of **1** and **4** are thus close to that of aromatic crosslinkers such as ethoxylated bisphenol-A dimethacrylate, EBPADMA (2 ethoxy groups, n^D^_20_ 1.525, η ≈ 900 mPa·s), but at a significantly lower viscosity and with the important difference, that all double bonds can take part in polymerization reactions. Compound **1** exhibits the most pronounced combination of a rather low viscosity and a high refractive index (Figure 1). Next, the solubility of the compounds **1–6** was tested in water, ethanol, isopropanol, acetone and methacrylic acid. Whereas all compounds **1–6** were highly soluble in acetone and methacrylic acid, no solubility in water was observed. A more differentiated analysis was possible using water/ethanol and water/isopropanol mixtures. By adding small amounts of alcohol to water (around 5% v/v), all compounds except **3** became fully soluble. Interestingly, compound **3** remained insoluble – even in pure ethanol or isopropanol – in contrast to compounds **1**, **2** and **4–6**. Overall, a broad solubility spectrum was found (Table 1).

**Table 1:** Solubility parameters of compounds **1–6**.^a^

compound	water	ethanol	isopropanol	acetone	methacrylic acid

**1**	−	+	+	+	+
**2**	−	+	+	+	+
**3**	−	−	−	+	+
**4**	−	+	+	+	+
**5**	−	+	+	+	+
**6**	−	+	+	+	+

^a^+ soluble in the respective solvent, − insoluble in the respective solvent.

### Polymerization behavior

**Figure 2:**
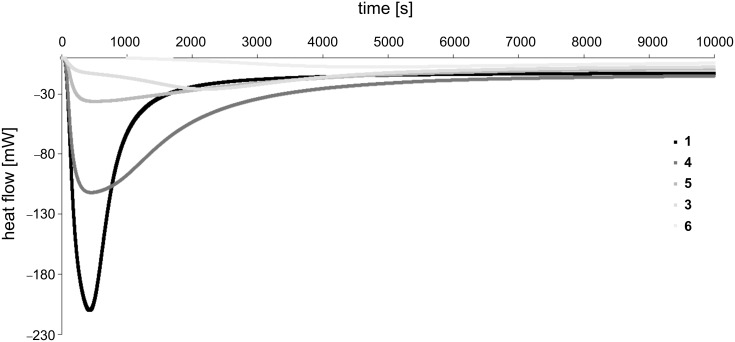
Exemplary photo-DSC plots for the curing of **1** and **3–6** at 37 °C.

**Figure 3:**
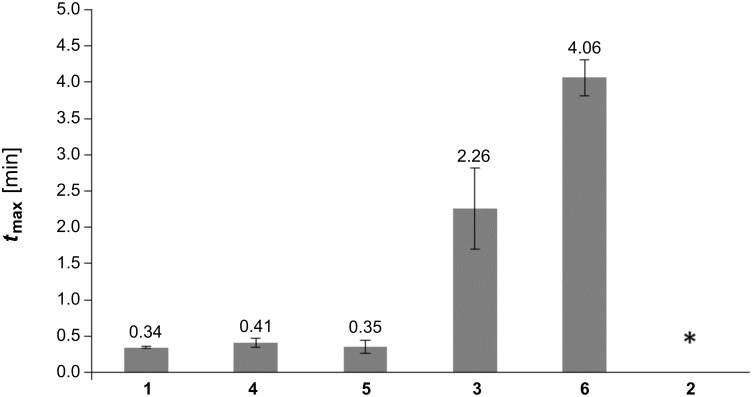
*t*_max_ for the curing of **1** and **3–6** at 37 °C (* no polymerization heat detected).

**Figure 4:**
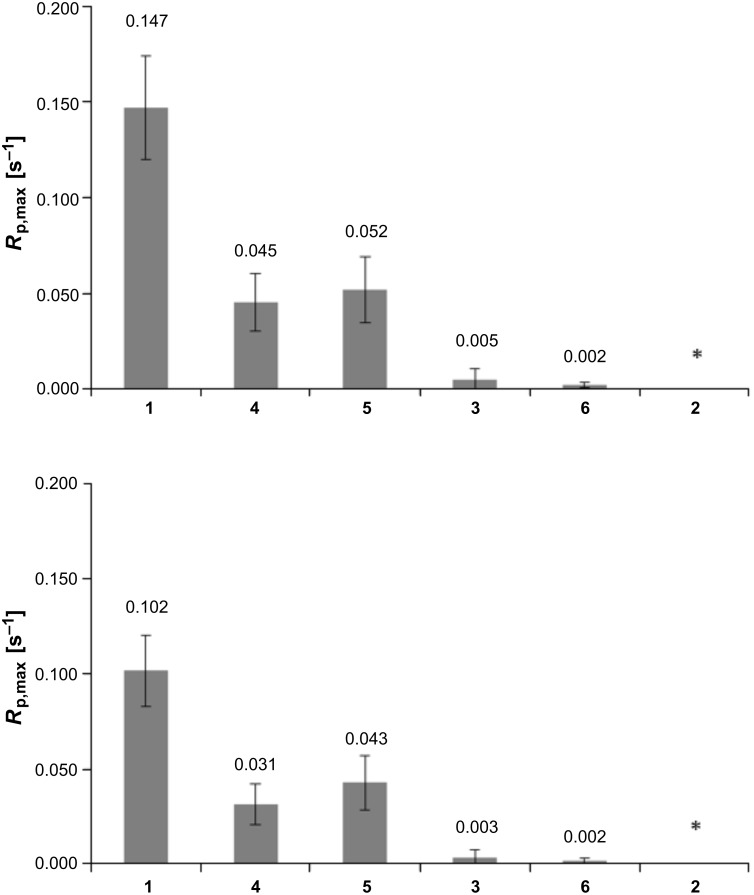
*R*_p, max_ for the curing of **1–6** at 37 °C for a) top: ∆*H*_p_ of allyl groups = 87.5 kJ·mol^−1^ and b) bottom: ∆*H*_p_ of allyl groups = 43.7 kJ·mol^−1^ (*no polymerization heat detected).

Bulk homopolymerization of **1** and **3–6** was monitored by photo-DSC. Curing plots showed a rapid polymerization for **1**, **4** and **5** (*t*_max_ = 20 s, 24 s and 21 s, respectively), while curing of **3** (*t*_max_ = 1 min 58 s) and **6** (*t*_max_ = 4 min 15 s) was delayed to later stages (Figure 2, Figure 3). As expected, irradiation of activated samples of **2** did not lead to any detectable polymerization heat at all, most likely due to obstructed allylic homopolymerization. As a) linear copolymerization of acrylamido and allyl moieties heavily favors acrylamide homopolymerization [28,32] and b) the allyl group is known for its chain-transfer behavior, but still c) reaction is fastest for the highest (intramolecular) occurrence of acryl/allyl groups, a dominant, non-classical polymerization mechanism of molecules containing both acrylamido and allyl functions can be assumed. The theoretical value for ∆*H*_p_ of the well-studied acrylamide-double bonds is 19.8 kcal·mol^−1^ (82.8 kJ·mol^−1^). For allyl double bonds, the disclosure is more complex. As mentioned, the tendency of allyl groups to homopolymerize is weak and in many cases, no polymerization can be observed at all. Therefore, we suggest a two-step estimation: In a best case, ∆*H*_p_ of allyl groups should be as high as that of 1-butene, which is 20.9 kcal·mol^−1^ (87.5 kJ·mol^−1^) [33]. In a worst case, the reactivity of allyl groups is only half of 1-butene’s reactivity, giving modest 10.5 kcal·mol^−1^ (43.7 kJ·mol^−1^). The borderline case that allyl groups would show no reactivity at all was not reflected in our calculations (however, it would result in the highest maximum polymerization rates, *R*_p,max_ values). Regarding R_p,max_ of **1** it stands out with a value of 0.147/0.102 (Figure 4). In case of compound **5** (0.052/0.043) and **4** (0.045/0.031) the values were slightly higher as or comparable to the often used 2-hydroxyethyl methacrylate (HEMA), as measured by a different work group (0.032) [34]. Interestingly, equipping the amide α-carbon with a methyl group (1→4) led to a ~70% decrease of *R*_p,max_ from 0.147/0.102 to 0.045/0.031, indicating the special role of the internal double bond. Also, when comparing **5** (0.052/0.043) and **3** (0.005/0.003), the internal allylic function contributes to a remarkable 10-fold higher *R*_p,max_ than both external allyl functions.

**Figure 5:**
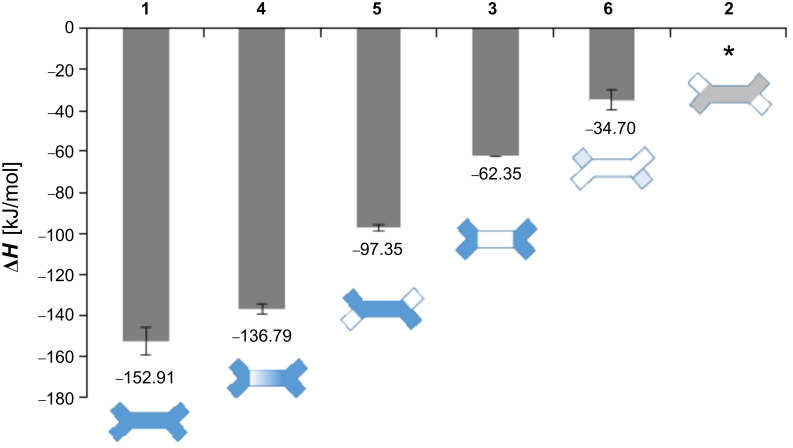
Polymerization heat, ∆*H*_p_ for the curing of **1–6** at 37 °C (* no polymerization heat detected).

The ∆*H*_p_ values ranged from ca. −35 to −153 kJ·mol^−1^ and, again, were highest for **1** (Figure 5). Notably, the ∆*H*_p_ of −153 kJ·mol^−1^ corresponds to almost two times the polymerization heat of primary acrylamide when fully converted (82.8 kJ·mol^−1^) [35]. However, as incomplete conversion under the tested bulk conditions has to be expected, spatially adjacent allyl groups have to take part in the polymerization. Interestingly, the internal allylic function again seems to contribute to a much higher extent to the overall reactivity when compared to two external allyl functions (**5** vs **3**).

**Figure 6:**
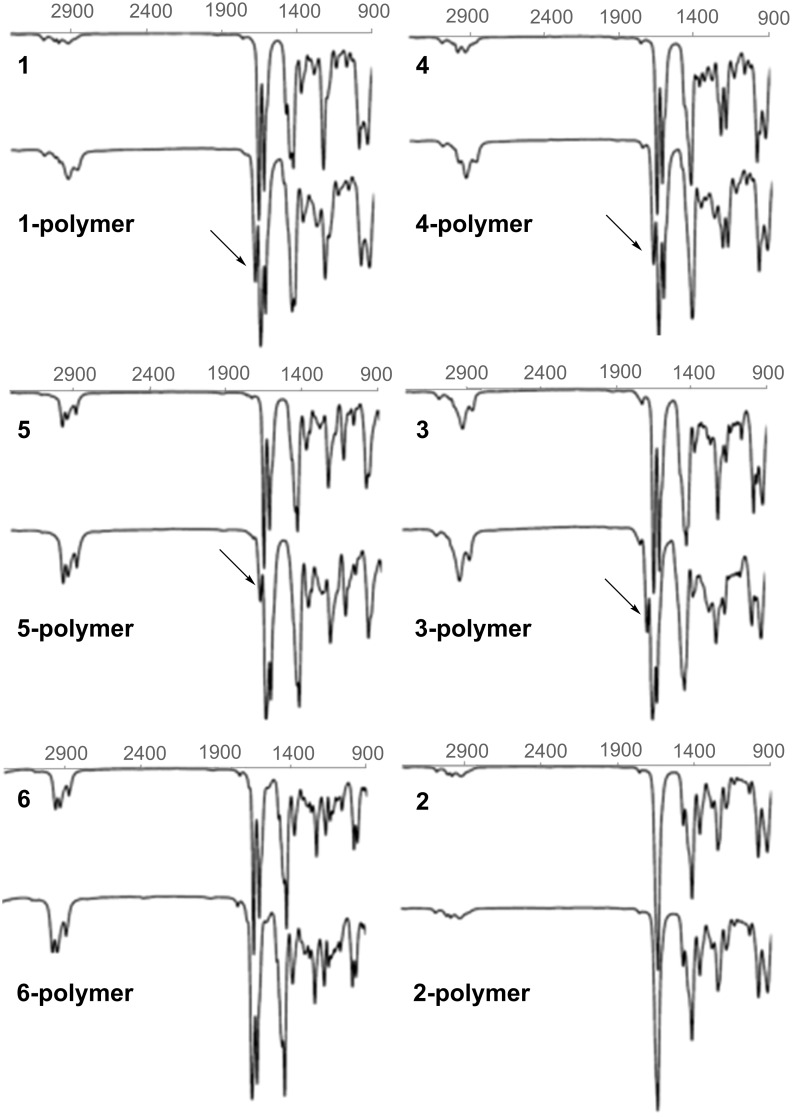
FTIR spectra of **1–6** before (top) and after (bottom) curing; the arrows indicate emerging, characteristic γ-lactam vibration at ~1680 cm^−1^ for polymers **1** and **3–5**.

**Scheme 6:**
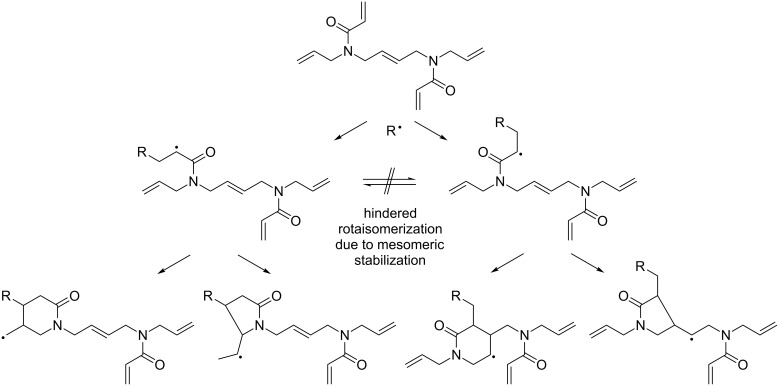
Proposed reaction pathways for the intramolecular propagation within **1**.

In order to verify an assumed, underlying cyclopolymerization mechanism, FTIR spectra of the crosslinkers were recorded before and after photopolymerization (Figure 6). All compounds containing both, acrylamido and allyl functions (**1**, **3–5**), showed two peaks for the acrylamide vibration at ≈1645 cm^−1^ and ≈1610 cm^−1^ before, and 3 peaks at ≈1645 cm^−1^, ≈1610 cm^−1^ and ≈1680 cm^−1^ after the polymerization. Compound **6** showed only two peaks before and after polymerization, while the spectrum of **2** contains the typical acetamide peak at 1633 cm^−1^ before and after polymerization. In accordance with the FTIR data and literature [27–28], we propose that some of the possible intramolecular reaction products (intermolecular cyclization products are conceivable as well) start most likely from the formed acrylamide radical as depicted in Scheme 6. Upon subsequent cyclization, either 6-membered δ-lactams, or 5-membered γ-lactams can be formed. The emerging FTIR signal at ≈1680 cm^−1^ strongly indicates the formation of γ-lactams as it can be attributed to the stretching vibration of γ-lactam carbonyl groups [28]. However, the corresponding δ-lactam peak could be located below the amide peak at ≈1645 cm^−1^. After one intramolecular ring is formed, there are three general options for a further radical reaction: a) Intermolecular radical propagation, b) intermolecular cyclization, and/or c) intramolecular cyclization. Assuming an incomplete conversion and judging from the remaining IR signals in the finger print regime, we can only assume that all three propagation pathways a), b) and c) take place simultaneously. Furthermore, taking in account FTIR and DSC data, we expect that ring formation significantly contributes to the overall reaction enthalpy. It is quite clear, that the combination of internal allylic and acrylamide functions is more favored than one of acrylamide functions with external allylic moieties. A reason for that might be the spatial arrangement of the rotationally obstructed double bond of the acrylamide group, which has two favorable out of three possible orientations to initiate the lactam formation with the internal butene group, whereas there is only one favorable orientation for the external allyl group.

### Polymer network properties

**Figure 7:**
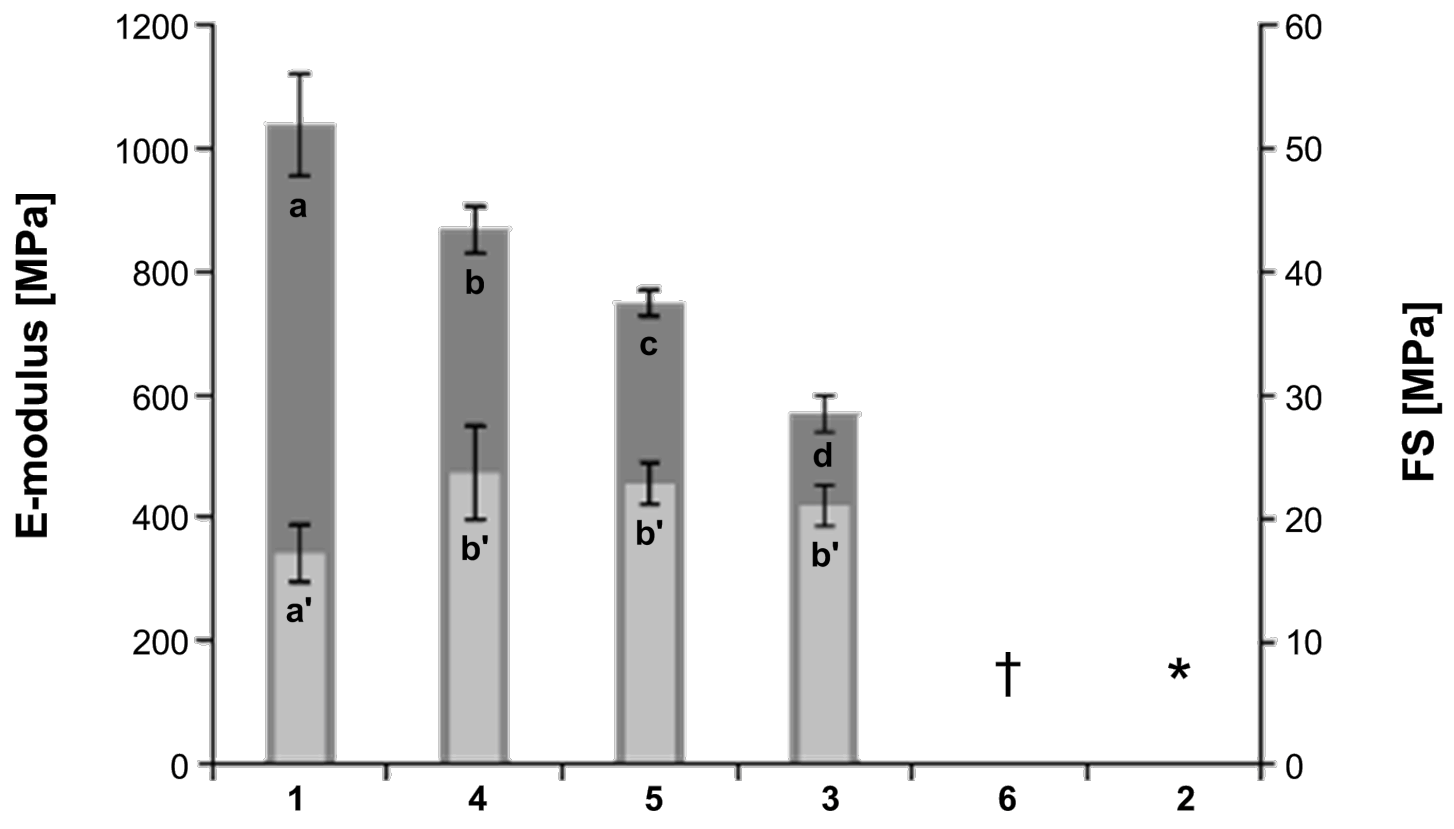
Flexural strength (FS) and E-modulus of cured crosslinkers **1**–**6**; letters refer to statistical groups (* gel-like polymer, pretest failure; † brittle polymer, pretest failure).

To assess the influence of functionalities on the network densities of the obtained polymers, mechanical data of rod-like samples according to ISO 4049 3-point bending was collected. The photocured sample of **2** was gel-like and could not be tested whereas compound **6** led to brittle material. Concerning the other samples, flexural moduli (E-moduli) were statistically different. The polymer of **1** exhibited the highest flexural-modulus and thus highest apparent network density. This means that ring formation, which in principle should reduce the amount of covalent network points in the cured material, is superimposed by effects of conversion, entanglement and rigidity of the formed polymer. The decreasing trend of flexural modulus from **1**, **4**, **5** to **3** is in accordance with the data on reactivity obtained from photo-DSC measurements (Figure 7).

## Conclusion

In this study, six polymerizable *N*,*N*’-diacylamides containing *N*-acryl, *N*-allyl and/or *N*-alkyl groups were synthesized in two steps. With the exception of the single solid *N*,*N*'-diacetyl-*N*,*N*'-diallyl-1,4-but-2-enediamine, all compounds were obtained as remarkably low-viscous liquids, characterized by high refractive indices above 1.500 and good solubilities in exemplary solvents. A significant increase in the polymerization reactivity and rate was achieved by systematic spatial intramolecular arrangement and the substitution with *N*-allyl, *N*-acyl and *N*-acrylamide functional groups. Surprisingly, the contribution of internal *N*-allyl groups was higher than that of external ones indicating a dominant, non-classical polymerization mechanism. The results support exothermic ring formation on the *N*,*N*’-diallyl-diacrylamide moieties being responsible for the considerable plus in reactivity. Due to the flexible character of the functional groups, intermolecular cyclization is also probable and thus, will contribute to the overall polymerization reactivity. A general building principle was found based on the synergistic combination of spatially adjacent functional groups. It allows a tremendous increase in the overall double-bond susceptibility in the studied molecules. The *N*,*N*'-diacryloyl-*N*,*N*'-diallyl-1,4-but-2-enediamine revealed the highest level of intramolecular synergism rendering this type of crosslinkers highly attractive for a broad range of free radical (photo)polymerization applications, for example in the constantly growing medical device sector.

## Experimental

**Materials.** Chemicals and reagents were purchased from Acros, Carl Roth, ChemPUR, Sigma-Aldrich, TCI or ABCR or have been used from the MCAT company stock and were used without further purification.

**Measurements.** TLC was carried out on Silica Gel 60 F254 (Merck, layer thickness 0.2 mm) with detection by UV light (254 nm) and/or by charring with 15% sulfuric acid in ethanol. Flash column chromatography (FC) was performed on M&N Silica Gel 60 (0.063–0.200 mm). ^1^H NMR and ^13^C NMR spectra were recorded on a Bruker Avance II 400. Chemical shifts are reported in ppm relative to solvent signals (CDCl_3_: δ_H_ = 7.26 ppm, δ_C_ = 77.0 ppm). Signals were assigned by first-order analysis and assignments were supported, where feasible, by two-dimensional ^1^H,^1^H and ^1^H,^13^C correlation spectroscopy. The polymerization enthalpies Δ*H* were measured with a photo-DSC 7/DPA 7 (Perkin-Elmer) having a light intensity in the visible portion of the spectrum of 108 mW/cm² in an isothermal mode at 37 °C. Each sample was activated with 0.3 wt % camphorquinone, 0.4 wt % ethyl 4-dimethylaminobenzoate and irradiated twice. After the first run a second run was made that was subtracted from the first one. The subtraction of these runs from one another removed the effect of sample heating by illumination. The DSC experiments were carried out twice and maximum rates of polymerization, *R*_p,max,_ were calculated according to Equation 1.

[1]
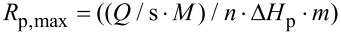


With *Q*/s being the heat flow per second at the global minimum of the first deviation of the respective measurement (maximum *Q*/s), *M* the molar mass of the monomer, *n* the number of double bonds per monomer molecule, ∆*H*_p_ the heat released per mole of double bonds reacted, and *m* the mass of the monomer in the sample. Mechanical data was measured in the 3-point bending mode according to ISO 4049:2009 using a Zwick instrument. Sample activation was done by adding 0.3 wt % camphorquinone and 0.4 wt % ethyl 4-dimethylaminobenzoate. FTIR spectra were measured using an iS10 FTIR spectrometer (Thermo Scientific). Viscosity was measured on an Anton Paar Physica MCR 300 equipped with CP 50-1 plate–plate geometry at a shear stress τ of 5 Pa and 25 data points from 0.72 to 1450 rad were taken and averaged. Refractive indices (RI) were measured using an Anton Paar Abbemat 200 refractometer at 20 °C.

### General procedure for the synthesis of *N*,*N*’-diacyl-*N*,*N*’-dialkyl-1,4-diamines **1–6**

#### Amination

a) Synthesis of 1,4-but-2-enediamine: Potassium carbonate (2.5 equiv) was added to the alkylamine (15 equiv) and cooled to 0–5 °C. The corresponding dibromide (1 equiv) was added in portions and the resulting mixture was stirred for 3–5 h at rt. Then the remaining amine was removed by distillation and the resulting residue was suspended in acetone. After removing the salts by filtration the acetone was evaporated.

b) Synthesis of the 1,4-butanediamine: The corresponding alkyl chloride (2.1 equiv) was added drop wise to a solution of 1,4-diaminobutane (1 equiv) in methanol at 50 °C. The resulting mixture was stirred at 60 °C for 24 h. Then methanol was removed by distillation and the residue was diluted with 2 M NaOH and extracted with DCM. The organic layer containing the crude product was dried (Na_2_SO_4_) and the solvent evaporated.

#### Acrylation

The resulting diamines were dissolved in THF and triethylamine (3.5 equiv) was added. Acryloyl chloride (2.2 equiv) was addeddrop wise at 0–5 °C after which the resulting mixture was stirred for 2.5 h at rt. Then, the THF was evaporated, ethyl acetate was added and the resulting mixture was washed 3 times with 2 N HCl and once with water. The organic layer was dried (Na_2_SO_4_), the solvent was evaporated and the residue was purified by flash chromatography.

***N*,*N*'-Diacryloyl-*N*,*N*'-diallyl-1,4-but-2-enediamine (1):** Yield: 45%; purity (^1^H NMR) >98%; η_23°C_ = 338 mPa·s; n^D^_20_ = 1.529; ^1^H NMR (CDCl_3_) δ 6.50–6.43 (m, 2H, *H*_2_CCHC(O)), 6.37–6.33 (m, 2H, *H*_2_CCHC(O)), 5.81–5.72 (m, 2H, H_2_CC*H*CH_2_), 5.69–5.66 (m, 2H, H_2_CC*H*C(O)), 5.59–5.56 (m, 2H, H_2_C*H*CC*H*CH_2_), 5.22–5.11 (m, 4H, *H*_2_CCHCH_2_), 4.02 (m, 4H, *H*_2_CHCCHC*H*_2_), 3.92 (m, H_2_CCHC*H*_2_); ^13^C NMR (CDCl_3_) δ 166.3, 166.1 (*C*(O)CHCH_2_), 132.9, 132.8 (H_2_C*C*HCH_2_), 128.5–127.3 (H_2_*CC*HC(O), H_2_CH*CC*HCH_2_), 117.5-116.7 (H_2_*C*CHCH_2_), 49.3–48.1 (H_2_CCH*C*H_2_), 47.1 (H_2_*C*HCCH*C*H_2_); FTIR 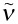
_max_ [cm^−1^]: 3517, 3080, 3018, 2986, 2917, 1645, 1611, 1463, 1416, 1363, 1276, 1213, 1129, 1059, 975, 919, 794.

***N*,*N*'-Diacetyl-*N*,*N*'-diallyl-1,4-but-2-enediamine (2):** Yield: 24%; purity (^1^H NMR) >97%; *T*_m_ = 32 °C; n^D^_20_ = 1.505; ^1^H NMR (CDCl_3_) δ 5.69–5.61 (m, 2H, H_2_CC*H*CH_2_), 5.43 (m, 2H, H_2_C*H*CC*H*CH_2_), 5.12–4.98 (m, 4H, *H*_2_CCHCH_2_) 3.88–3.74 (m, 8H, *H*_2_CHCCHC*H*_2_, H_2_CCHC*H*_2_), 2.00 (s, 6H, C(O)C*H*_3_); ^13^C NMR (CDCl_3_) δ 170.3–169.4 (*C*(O)CH_3_), 132.9–132.3 (H_2_C*C*HCH_2_), 127.9–127.0 (H_2_CH*CC*HCH_2_), 116.7–116.2 (H_2_*C*CHCH_2_), 49.9–46.2 (H_2_CCH*C*H_2_, H_2_*C*HCCH*C*H_2_) 21.1–21.0 (C(O)*C*H_3_); FTIR 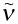
_max_ [cm^−1^]: 3074, 3012, 2986, 2916, 1633, 1468, 1411, 1360, 1242, 1187, 1035, 978, 919.

***N*,*N*'-Diacryloyl-*N*,*N*'-diallyl-1,4-butanediamine (3):** Yield: 35%; purity (^1^H NMR) >94%; η_23°C_ = 382 mPa·s; n^D^_20_ = 1.515; ^1^H NMR (CDCl_3_) δ 6.58–6.28 (m, 4H, *H*_2_CCHC(O)), 5.80–5.71 (m, 2H, H_2_CC*H*CH_2_), 5.68–5.60 (m, 2H, H_2_CC*H*C(O)), 5.21–5.10 (m, 4H, *H*_2_CCHCH_2_), 4.02–3.93 (m, 4H, H_2_CCHC*H*_2_), 3.41–3.40 (m, 4H, *H*_2_CH_2_CCH_2_C*H*_2_), 1.55 (m, 4H, H_2_C*H*_2_CC*H*_2_CH_2_); ^13^C NMR (CDCl_3_) δ 166.6, 166.0 (*C*(O)CHCH_2_), 133.3, 133.0 (H_2_C*C*HCH_2_), 128.2–127.5 (H_2_*CC*HC(O)), 117.1–116.7 (H_2_*C*CHCH_2_), 50.1–48.6 (H_2_CCH*C*H_2_), 46.9–45.9 (H_2_*C*H_2_CCH_2_*C*H_2_), 26.5–25.0 (H_2_CH_2_*CC*H_2_CH_2_); FTIR 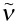
_max_ [cm^−1^]: 3472, 3082, 2924, 1646, 1609, 1428, 1374, 1217, 1163, 1133, 1059, 978, 957, 918, 794.

***N*,*N*'-Diacryloyl-*N*,*N*'-diallyl-2,4-pent-2-enediamine (4):** Yield: 14%; purity (^1^H NMR) >98%; η_23°C_ = 409 mPa·s; n^D^_20_ = 1.526; ^1^H NMR (CDCl_3_) δ 6.50–6.26 (m, 5H, 2x *H*_2_CCHC(O)), 5.84–5.70 (m, 2H, H_2_CC*H*CH_2_), 5.68–5.60 (m, 2H, H_2_CC*H*C(O)), 5.61–5.51 (m, 2H, H_2_C*H*CCHCH_2_), 5.31 (m, 1H, *H*C(CH_3_)HCCHCH_2_), 5.25–5.08 (m, 4H, *H*_2_CCHCH_2_), 4.06–3.71 (m, 6H, HC(CH_3_)HCCHC*H*_2_, 2× H_2_CCHC*H*_2_); ^13^C NMR (CDCl_3_) δ 166.4, 166.3, 166.1 (*C*(O)CHCH_2_), 135.1–132.8 (H_2_C*C*HCH_2_), 128.5–126.5 (H_2_*CC*HC(O), HC(CH_3_)H*CC*HCH_2_), 117.5–116.4 (H_2_*C*CHCH_2_), 50.0 (H*C*(CH_3_)HCCHCH_2_), 49.1–45.5 (H_2_CCH*C*H_2_, H_2_*C*HCCHCH_2_), 18.7, 17.1, 16.8 (HC(*C*H_3_)HCCHCH_2_); FTIR 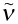
_max_ [cm^−1^]: 3532, 3491, 3080, 2977, 2924, 1644, 1609, 1416, 1362, 1328 1276, 1217, 1184, 1129, 1059, 976, 919, 794.

***N*,*N*'-Diacryloyl-*N*,*N*'-dipropyl-1,4-but-2-enediamine (5):** Yield: 33%; purity (^1^H NMR) >97%; η_23°C_ = 428 mPa·s; n^D^_20_ = 1.5095; ^1^H NMR (CDCl_3_) δ 6.54–6.47 (m, 2H, *H*_2_CCHC(O)), 6.33–6.25 (m, 2H, *H*_2_CCHC(O)), 5.66–5.58 (m, 2H, H_2_CC*H*C(O)), 5.56–5.51 (m, 2H, H_2_C*H*CC*H*CH_2_), 3.99–3.89 (m, 4H, *H*_2_CHCCHC*H*_2_), 3.30–3.18 (m, H_3_CCH_2_C*H*_2_), 1.54 (‘quint’, 4H, H_3_CC*H*_2_CH_2_), 0.85 (t, 6H, *H*_3_CCH_2_CH_2_); ^13^C NMR (CDCl_3_) δ 166.1, 165.9 (*C*(O)CHCH_2_), 128.1–127.3 (H_2_*CC*HC(O), H_2_CH*CC*HCH_2_), 117.5–116.7 (H_2_*C*CHCH_2_), 49.1–47.4 (H_3_CCH_2_*C*H_2_, H_2_*C*HCCH*C*H_2_), 22.5–20.9 (H_2_CH_2_*CC*H_2_CH_2_, H_3_C*C*H_2_CH_2_), 11.2–11.0 (H_3_*C*CH_2_CH_2_); FTIR 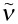
_max_ [cm^−1^]: 3525, 2963, 2932, 2875, 1645, 1609, 1442, 1426, 1368, 1279, 1224, 1123, 1059, 975, 888, 794.

***N*,*N*'-Diacryloyl-*N*,*N*'-dipropyl-1,4-butanediamine (6):** Yield: 26%; purity (^1^H NMR) >96%; η_23°C_ = 486 mPa·s; n^D^_20_ = 1.515; ^1^H NMR (CDCl_3_) δ 6.40–6.33 (m, 2H, *H*_2_CCHC(O)), 6.16–6.09 (m, 2H, *H*_2_CCHC(O)), 5.49–5.43 (m, 2H, H_2_CC*H*C(O)), 3.21–3.05 (m, 8H, *H*_2_CH_2_CCH_2_C*H*_2_, H_3_CCH_2_C*H*_2_), 1.38 (m, 8H, H_3_CC*H*_2_CH_2_, H_2_C*H*_2_CC*H*_2_CH_2_), 0.70 (m, H_3_CC*H*_2_CH_2_); ^13^C NMR (CDCl_3_) δ 165.5, 165.4 (*C*(O)CHCH_2_), 127.5–126.9 (H_2_*CC*HC(O)), 49.1–45.3 (H_3_CCH_2_*C*H_2_, H_2_*C*H_2_CCH_2_*C*H_2_), 26.3–20.5 (H_2_CH_2_*CC*H_2_CH_2_, H_3_C*C*H_2_CH_2_), 10.9–10.6 (H_3_*C*CH_2_CH_2_); FTIR 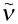
_max_ [cm^−1^]: 3314, 2963, 2933, 2874, 1645, 1608, 1481, 1449, 1426, 1374, 1263, 1227, 1166, 1136, 1058, 978, 954, 794.
